# Influence of health literacy on health outcomes of different social strata—— an empirical study based on the data of China's health literacy investigation

**DOI:** 10.1186/s12939-023-01858-x

**Published:** 2023-03-10

**Authors:** Huifang Yu, Siwen Sun, Jie Ling, Haixiao Chen, Guilin Liu

**Affiliations:** 1 Jiaxing Center for Disease Control and Prevention, Jiaxing, Zhejiang China; 2 School of Public Health and Management, Wenzhou Medical University, Wenzhou, Zhejiang China; 3 Taizhou Hospital of Zhejiang Province, Wenzhou Medical University, Taizhou, Zhejiang China

**Keywords:** Health literacy, Heath outcomes, Health disparities, Social stratum, Socioeconomic status

## Abstract

**Background:**

Health literacy has always been considered as an important factor to promote people's health, but does it have a significant effect on health across all social strata and especially lower social strata? This study aims to analyze the influences of health literacy on health outcomes of different social strata, and then infer whether improving health literacy can reduce health disparities among different social strata.

**Methods:**

Utilizing health literacy monitoring data from a city in Zhejiang Province in 2020, the samples are divided into three social strata according to the socioeconomic status score: low, middle and high social stratum, to compare whether there are significant differences in health outcomes between population with lower and higher health literacy among different social strata. In the strata with significant differences, control the confounding factors to further verify the influence of health literacy on health outcomes.

**Results:**

In low and middle social strata, there are significant differences between population with lower and higher health literacy, when considering the two types of health outcomes (chronic diseases and self-rated health), but in high social stratum, this difference is not significant. After controlling the relevant variables, the influence of health literacy on the prevalence of chronic diseases is statistically significant only in low social stratum, and the health literacy is negatively correlated with the prevalence of chronic diseases(OR = 0.722, *P* = 0.022). In addition, there are statistical significances for positive impact of health literacy on self-rated health in both low and middle social strata (OR = 1.285, *P* = 0.047; OR = 1.401, *P* = 0.023).

**Conclusion:**

Compared with high social stratum, the influence of health literacy on health outcomes of low social stratum (chronic diseases) or both middle and low social strata (self-rated health) is more significant, and both are to improve the health outcomes. This finding suggests that improving residents' health literacy may be an effective way to alleviate the health disparities among different social strata.

## Introduction

Since the Black Report was published in 1980 [1], the health disparities among different social strata have gradually attracted the attention of scholars from all countries. Most studies have found that the health status of low social stratum is often worse [2, 3]. With the development of medical technology and the continuous promotion of public health measures, the life expectancy of people in all countries around the world has been increasing, but the health disparities among different social strata still exist and even tend to get worse. Ten years after the Black Report was published, Smith and other scholars conducted another survey in British society and found that the health disparities among different British social strata were still expanding [4], and similar findings were found in the studies conducted by Tetzlaff and Fors [5, 6].

In order to alleviate this phenomenon of health inequality, researchers began to look for the reasons why health disparities exist among different social strata. In the field of health and medicine, some researchers believe that one of the important reasons for the emergence and continuous expansion of such health disparities is the uneven distribution of benefits brought by the progress of medical technology and various health promotion policies and measures in the whole society [7, 8]. For example, Pavalko believed that the advantages and resources possessed by people with higher socioeconomic status will make it easier for them to access and utilize new health promotion mechanisms, which resulted in population with high socioeconomic status would benefit more, while the poorest and the least educated population will benefit least. In order to reduce the uneven distribution of benefits among different social strata, governments and academia in all countries have begun to taken measures to improve the health status of low social stratum. Most of the measures are committed to providing a healthy supportive environment for population with low socioeconomic status, so that they have "the opportunity" to make healthy choices, such as establishing medical insurance systems, basic public health service systems, medical resources sinking and other measures [9, 10]. However, besides a few mandatory measures, most of the health services need residents to actively participate and utilize, especially the cultivation of a healthy lifestyle needs long-term self-consciousness. Compared with the population with high socioeconomic status, the population with low socioeconomic status often lack the ability to acquire, distinguish and utilize health information and health services [11–13], which is just the manifestation of lack of health literacy. The existing researches also indicate that the level of health literacy^1^ of the population with low socioeconomic status is generally low [14].

Health literacy is defined as " The degree to which individuals have the capacity to obtain, process, and understand basic health information and services needed to make appropriate health decisions "[15]. The basic differences between improving health literacy and other public health measures lie in that it is an internalized process, and its purpose is to enable people to make healthy choices sincerely, voluntarily and willingly. It can be believed that improving health literacy is the internal driving force for other public health measures to play their role. Therefore, analyzing influence of health literacy on health outcomes plays an irreplaceable role in finding ways to reduce the health disparities among different strata.

The current researches on the relationship between health literacy and health outcomes can be roughly divided into the following aspects according to different health outcomes: (1) The influence on disease prevalence and prognosis. For example, the population with low health literacy have a higher prevalence of chronic diseases [16], and perform worse in disease control and complication prevalence [17]. (2) Influence on mortality. Lower health literacy is associated with higher mortality [18]. (3) Influence on self-rated health status. The self-rated health status of the population with low health literacy is worse [19, 20]. Most of these studies are conducted in the whole population or divided into different subgroups according to gender, age and other characteristics, respectively studying the influence of health literacy on health outcomes in each subgroup. However, few studies have considered social strata of samples according to their socioeconomic status to understand the relationship between health literacy and health outcomes in different social strata. Although health literacy plays a positive role in promoting health for most health outcomes in the study of the whole population, it is uncertain whether health literacy can also play a positive role in different social strata, especially in low social stratum. If we want to alleviate the health disparities among different social strata by improving health literacy, we must first understand the influence of health literacy on health outcomes in all social strata, and then further infer whether this method to improving health literacy can play desired role in reducing health disparities among different social strata.

In this study, three classical measurement indicators, education level, income level and professional status [21], are used to measure socioeconomic status, the scores of health literacy questionnaire are used to measure whether the samples have health literacy, and the prevalence of chronic diseases and self-rated health status are used as indicators to measure health outcomes. According to the score of socioeconomic status, the samples are divided into three social strata: low, middle and high social stratum, and the influence of health literacy on health outcomes in different social strata is analyzed, so as to provide scientific evidences to find effective ways to reduce the health disparities among different social strata.

## Methods

### Respondent

This survey is part of a survey of residents' health literacy in Zhejiang Province conducted by the Zhejiang Center for Disease Control and Prevention. The respondents were found in seven counties of a city in Zhejiang Province. Residents aged 15–69 who had lived in the local area for more than 6 months totally from July 2019 to June 2020 are selected as the respondents, but do not include residents who collectively lived in hospitals, dormitories, nursing homes, etc.

### Research methods

#### Sampling method

**Fig. 1:**
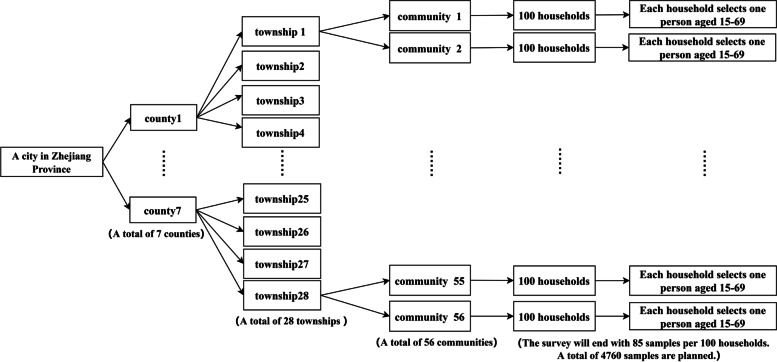
Sampling flow chart

The samples are selected by stratified multistage random sampling. In the first stage, four townships are randomly selected from each county, and a total of 28 townships surveyed places are selected. In the second stage, two communities are randomly selected from each township. In the third stage, 100 households are selected from each community, and one resident aged 15–69 is selected from each household as the respondent. It is enough to complete 85 questionnaires in each community, and a total of 4,760 samples are obtained (Fig. 1). In this study,we excluded respondents aged 15 to 17. Because we need to know the professions of the respondents to measure people's socioeconomic status, yet most respondents did not work before the age of 18. After initial screening according to age, there are 4,693 respondents aged between 18 and 69.

#### Survey method

In this survey, the method of questionnaire survey and household survey are both adopted. The questionnaire is completed by the respondents. If the respondents can not complete the questionnaire independently, it will be surveyed by face-to-face inquiry. Before the survey, investigators were trained standardly to ensure the consistency of survey method used. During the survey, the on-site coordinators will supervise and verify whether the investigators comply with the survey technical specifications. After the survey, the disease control department will conduct quality control by checking the answer time in the system background, extracting sound recordings and on-site review, exclude the unqualified questionnaires, and select new respondents again, so as to obtain all the qualified data finally.

The questionnaire includes three parts: General information survey, Health literacy survey and Health status survey. The general information survey mainly collects the age, gender, profession, education level, income level and other information of the respondents. Health literacy was assessed by the Chinese Citizen Health Literacy Questionnaire, which was designed by Delphi method [22]. Experts in the fields of public health, health education and promotion, and clinical medicine jointly designed this questionnaire. And the respondents of this questionnaire are permanent urban and rural residents aged 15–69 in China. The overall Cronbach’s alpha of the questionnaire was 0.95 and Spearman-Brown coefficient was 0.94 [23]. This questionnaire is not only used in the annual China Health Literacy Survey (CHLS) [24], but also in many studies on health literacy in China [25–27]. The health status survey part is used to investigate the health outcomes of recent chronic diseases and self-rated health status.

#### Statistical method

SPSS 22.0 is used for statistical analysis. Because the variables in this study are categorical variables, the categorical variables are expressed as constituent ratio (%) in statistical description, and the chi-square test is adopted for the inter-group difference test. Logistic regression model is used to further determined the influence of health literacy on health outcomes, and the significance level is set at α = 0.05.

### Assignment standard

Use profession status, education level and income level to measure the socioeconomic status of the respondents. As for which of the three variables of education, income and occupation is more important, the opinions of various researchers are not consistent [28, 29], so this paper still adds these three variables with equal weight [30]. The health literacy questionnaire included three types of questions: true/false (correct response received 1 points), single-answer (correct response received 1 points), and multiple-answer (correct responses received 2 points). The total score of the health literacy questionnaire is 66, and those who reach 80% or more of the total score are judged to have basic health literacy [31]. Chronic disease and self-rated health are selected as indicators to measure health status. See Table 1 for specific assignment standards of each variable.

**Table 1:** Variable definition and assignment

Variable	Definition and assignment
Education level	1 = Illiterate/Primary school, 2 = Junior high school, 3 = Senior high school/Vocational high school/Technical secondary school, 4 = Junior college/University, 5 = Postgraduate and higher
Income level	Annual per capita household income = Total annual household income/Household size. 1 = Less than 10,000 yuan; 2 = 10,000–29,999 yuan; 3 = 30,000–49,999 yuan; 4 = 50,000–69,999 yuan; 5 = 70,000 yuan and higher
Professional status	1 = The unemployed/Retiree; 2 = Farmer/Worker; 3 = Enterprise employee/Personnel of other public institutions/Businessman/College student; 4 = Teacher/Medical staff; 5 = Civil servant
Socioeconomic status	The individual's comprehensive socioeconomic status is measured by adding the scores of education level, income level and professional status. The higher the score is, the higher the status is. The actual lowest score in all samples is 4 and the highest score is 14. 4–7 of socioeconomic status score = Population with low socioeconomic status, 8–10 = Population with middle socioeconomic status, 11–14 = Population with high socioeconomic status
Chronic disease	1 = Suffering from any one or more chronic diseases, such as hypertension, diabetes, cerebrovascular disease, etc.; Otherwise = 0
Self-rated health	1 = Self-rated health is "good" or "better"; Otherwise = 0

## Result

### General information of respondents

Four thousand six hundred ninety-three samples are screened by logical test and outlier cleaning, and 4011 valid questionnaires are obtained, with an effective rate of 85.47%. Descriptive statistical analysis is made on 4011 valid samples after screening, and the general information is as follows: 1,981 males, accounting for 49.4%, and 2,030 females, accounting for 50.6%; The age distribution is dominated by middle-aged people aged 40–59, accounting for 47.8%, young people aged 18–39, accounting for 30.0%, and elderly people aged 60–69, accounting for 22.1%; The marital status is mainly married, accounting for 84.0%; Census register is dominated by local census register, accounting for 88%; After the sample is stratified according to the socioeconomic status score, the low, middle and high levels account for 61.6%, 28.5% and 9.9% respectively; The population with higher health literacy accounts for 31.2% of the total sample. See Table 2 for details.

**Table 2:** General information of respondents (*N* = 4011)

Variable	Frequency	Percentage%
**Gender**		
Male	1981	49.4
Female	2030	50.6
**Age**		
18 ~ 39 years	1204	30.0
40 ~ 59 years	1919	47.8
60 ~ 69 years	888	22.1
**Rural/Urban**		
Rural	2416	60.2
Urban	1595	39.8
**Marital Status**		
Single	406	10.1
Married	3369	84.0
Separated	21	0.5
Divorced	120	3.0
Widowed	95	2.4
**Census register**		
Local	3530	88.0
Other places	481	12.0
**Socioeconomic status**		
Low	2471	61.6
Middle	1142	28.5
High	398	9.9
**Health literacy**		
Lower	2761	68.8
Higher	1250	31.2

### Differences in health outcomes and health literacy among different social strata

The statistical results in Table 3 show that there are significant differences between the two types of health outcomes and health literacy among different social strata. The prevalence of chronic diseases is 34.4%, 16.3% and 7.5% in low, middle and high social stratum respectively. The proportion of self-rated health as good and better in low social stratum is 65.5%, while that in middle and high social strata is 75.5% and 80.7% respectively. There is a significant stratum gradient in the prevalence of chronic diseases and self-rated health status of different social strata. There are also stratum differences in health literacy. The health literacy level of low social stratum is 15.3%, which is significantly lower than that in middle and high social strata (49.1% and 78.1%).

**Table 3:** Differences in health outcomes and health literacy among different social strata

Socioeconomic status	Health outcomes				Health literacy level	
	**Chronic diseases**		**Good self-rated health status**			
	%	$\chi$^2^	%	$\chi$^2^	%	$\chi$^2^
Low	34.4	211.698^**^	65.5	61.646^**^	15.3	871.128^**^
Middle	16.3		75.5		49.1	
High	7.5		80.7		78.1	

^**^p < 0.01

### Differences in health outcomes between population with lower and higher health literacy in stratified samples

Based on the significant differences in health literacy and health outcomes among different social strata, it is speculated that the influence of health literacy on health outcomes may be different among different social strata. To test this hypothesis, in this study, whether there are differences between the two types of health outcomes in population with lower and higher health literacy in different social strata are compared at first (Table 4). The results show that there are significant differences in the prevalence of chronic diseases and self-rated health status between population with lower and higher health literacy in low and middle social strata. In high social stratum, although the prevalence of chronic diseases in population with higher health literacy is slightly lower than that in population with lower health literacy and the self-rated health status is better, the difference is not statistically significant. Therefore, it can't be considered that there is a difference in the two types of health outcomes between population with lower and higher health literacy in high social stratum.

**Table 4:** Differences in health outcomes between population with lower and higher health literacy in stratified samples

Socioeconomic status	Health literacy	Chronic diseases		Good self-rated health status	
		%	$\chi$^2^	%	$\chi$^2^
Low	Lower	36.6	28.238^**^	64.3	8.285^**^
	Higher	22.5		72	
Middle	Lower	22.4	31.098^**^	71.3	11.409^**^
	Higher	10.2		79.9	
High	Lower	9.2	0.439	77	0.946
	Higher	7.1		81.7	

^**^p < 0.01

### Influences of health literacy on health outcomes in stratified samples

In order to further verify the relationship between health literacy and health outcomes found in low and middle social strata, multivariate logistic regression analyses of health literacy and chronic diseases or self-rated health in low and middle social strata are conducted respectively. Before regression analysis, difference test is performed to determine confounders that might affect health outcomes (Table 5).

**Table 5:** Difference test for health outcomes in populations with different characteristics

Variable	Low socioeconomic status				Middle socioeconomic status			
	**Chronic diseases**		**Good or better self-rated health status**		**Chronic diseases**		**Good or better self-rated health status**	
	%	$\chi$^2^	%	$\chi$^2^	%	$\chi$^2^	%	$\chi$^2^
**Gender**								
Male	38.1	13.572^**^	67.2	3.009	20.2	15.096^**^	76.9	1.384
Female	31		63.9		11.8		73.9	
**Age**								
18 ~ 39 years	4.0	320.986^**^	79.1	32.356^**^	3.3	226.308^**^	80.1	20.011^**^
40 ~ 59 years	28.3		65.2		25.6		72.1	
60 ~ 69 years	57.1		60.7		57.6		61.6	
**Rural/Urban**								
Rural	33.8	0.913	64.6	1.499	15.2	1.571	74.3	1.14
Urban	35.7		67.0		18.0		77.1	
**Marital Status**								
Single	24.4	9.289	62.2	1.419	3.0	56.571^**^	85.2	20.037^**^
Married	34.5		65.7		19.5		73.4	
Separated	40.0		70.0		14.3		85.7	
Divorced	24.2		65.7		15.9		61.4	
Widowed	45		60.0		61.5		76.9	
**Census register**								
Local	38.1	106.331^**^	63.5	29.392^**^	17.9	13.999^**^	74.9	1.689
Other places	8.0		79.4		5.2		80.0	

^**^p < 0.01

The difference test results show that, in low social stratum, there are significant differences in the prevalence of chronic diseases among different genders, ages and census registers, and in self-rated health status in different ages and census registers. In middle social stratum, there are significant differences in the prevalence of chronic diseases among different genders, ages, marital statuses and census registers, and in self-rated health status among different ages and marital statuses. These variables, which may affect the prevalence of chronic diseases and self-rated health status, are included as control variables in the regression model with health literacy as independent variable and chronic diseases or self-rated health are included as dependent variable (Table 6).

**Table 6:** Analysis of the relationship between health literacy and health outcomes in different social strata

Variable	OR (Chronic disease)		OR (Self-rated health)	
**Socioeconomic status**	Low	Middle	Low	Middle
**Constant**	0.082^**^	0.044^**^	2.732^**^	4.759^**^
**Health literacy(ref. Lower)**				
Higher	0.722^*^	0.790	1.285^*^	1.401^*^
**Gender (ref. Male)**				
Female	0.760^**^	0.583^**^		
**Age (ref. 18 ~ 39 years)**				
40 ~ 59 years	6.386^**^	7.855^**^	0.626^**^	0.850
60 ~ 69 years	19.238^**^	26.967^**^	0.550^**^	0.521^*^
**Marital Status(ref. Single)**				
Married		1.422		0.581^*^
Live apart		0.847		1.545
Divorced		1.204		0.327^**^
Widowed		2.951		1.102
**Census register(ref. Local)**				
Other places	0.305^**^	0.433^*^	1.760^**^	
Likelihood ratio test	*P* < 0.001	*P* < 0.001	*P* < 0.001	*P* < 0.001
Hosmer–Lemeshow test	*P* = 0.221	*P* = 0.353	*P* = 0.886	*P* = 0.536

^*^p < 0.05

^**^p < 0.01

The validity test results of models of health literacy and two kinds of health outcomes are shown in Table 6. All models have passed the Likelihood ratio test (*P* < 0.05) and Hosmer–Lemeshow test (*P* > 0.05). The fitting results of each regression model are good.

After stratification, it can be observed that the risk of chronic diseases in population with higher health literacy is lower than that in population with lower health literacy. It can be considered that, for low social stratum, having higher health literacy can reduce the risk of chronic diseases(OR = 0.722, *P* = 0.022). However, in the middle socioeconomic stratum, after controlling other related variables, the influence of health literacy on chronic diseases is no longer statistically significant.

The positive influence of health literacy on self-rated health status is statistically significant in low and middle socioeconomic strata (OR = 1.285, *P* = 0.047; OR = 1.401, *P* = 0.023). For low and middle social strata, having higher health literacy is helpful to improve self-rated health status.

## Discussion

### Influence of health literacy on health outcomes of low social stratum (chronic diseases) or low and middle social strata (self-rated health) is more significant than that of high social stratum.

The difference of chronic disease prevalence and self-rated health status between population with lower and higher health literacy is only significant in low and middle social strata. After controlling the related variables, the influence of health literacy on chronic diseases is still statistically significant in population with low socioeconomic status, but such significant influence is not found in population with middle and high socioeconomic status. The influence of health literacy on self-rated health is statistically significant in population with low and middle socioeconomic status, but the correlation between health literacy and self-rated health is not found in population with high socioeconomic status.

Based on these results, it can be preliminarily inferred that the influences of health literacy on the health outcomes in low social stratum (chronic diseases) or low and middle social strata (self-rated health) is more significant than that in high social stratum, which is similar to the results of research conducted by Gibney in Ireland [32]. Gibney found that the influence of health literacy on health outcomes, such as chronic diseases and hospital attendance rate, was significant in low or middle and low social strata, but not in high social stratum. However, he did not explain detailly for this finding.

In this paper, we will attempt to explain this phenomenon from the following perspectives: Some researchers have found that the population that people come into contact with in work and life are mostly people in similar social stratum [33]. Because people have social needs, they are often imperceptibly influenced by the values and behavioral norms of surrounding people [34]. Population with high socioeconomic status have a high level of health literacy (78.1%). Even if population with high social stratum have not health literacy, they will still be influenced and restrained by the surrounding people and environment, which will encourage them to maintain a healthy lifestyle. In addition, most of the population with high socioeconomic status have a good living and working environment, and their chances to be exposed to the risk factors affecting their health are less [35, 36],which also weakens the role of health literacy to some extent. However, in low social stratum, the proportion of people with health literacy is very low (15.3%), and they are more likely to be exposed to health risk factors than those with high socioeconomic status. Therefore, health literacy has a greater influence on the health outcomes of population in low social stratum.

### Having higher health literacy can improve health outcomes (chronic diseases, self-rated health)

Among the significant influences of health literacy on health outcomes found in low and middle social strata, health literacy will all play a role to improve health outcomes. Those with higher health literacy had lower rates of chronic disease and better self-rated health status than those with lower health literacy. It is consistent with previous findings [16, 25].

People with higher health literacy are more willing and able to acquire and understand health knowledge, and utilize it to improve their lifestyle. However, one of the important reasons for chronic diseases and many other health damage is the long-term accumulation of health damage caused by unhealthy lifestyles [37]. In addition, Parikh believed that people with lower health literacy were easy to feel ashamed and embarrassed about their ignorance, which would hinder them from seeking health help including medical care services and acquisition of health knowledge, thus affecting their health status [38]. These findings can explain the improvement of health literacy on health outcomes to some extent.

### There are significant differences in health outcomes among different social strata

There is a significant stratum gradient in the prevalence of chronic diseases and self-rated health status among low, middle and high social strata. The prevalence of chronic diseases in population with low socioeconomic status (34.4%) is significantly higher than that in population with middle and high socioeconomic status (16.3%,7.5%), and their proportion of self-rated health as good or better (65.5%) is significantly lower than that in middle and high social strata (75.5%,80.7%). This is consistent with many study conclusions. For example, Roberto found that in almost all of the 22 European countries he surveyed, socioeconomic status had a significant negative correlation with the mortality rate and self-rated health [2]; Wolff believed that low subjective social status was significantly related to poor/common health status [39]. The same findings are found in the researches conducted in China [40, 41]. Generally speaking, the lower the socioeconomic status is, the worse the health status is. There are many reasons leading to health disparities among different social strata, including poor living and working environment [42], unhealthy lifestyle [37] and unequal access to medical resources [43] of population with low socioeconomic status, and the lack of health drive force caused by the insufficient health literacy discussed in this paper.

Combining conclusion 1 and 2, it can be found that health literacy has a more significant influence on health outcomes of population in low social stratum than that in high social stratum, and health literacy is a protective factor for health outcomes. Therefore, it can be considered that improving residents' health literacy is an effective measure to alleviate the health gap among social strata. While the government is committed to creating a healthy supportive environment for population with low socioeconomic status and improving the fairness of medical resources, it should also pay attention to the improvement of residents' health literacy, so that population of low social stratum not only have the opportunity but also have the ability to make healthy choices.

### Conclusions and shortcomings

In this paper, the influences of health literacy on health outcomes in all population with different socioeconomic status are discussed, according to the monitoring data of health literacy from a city of Zhejiang Province in 2020. The main conclusions are as follows: (1) The influences of health literacy on health outcomes of population in low social stratum (chronic diseases) or low and middle social strata (self-rated health) is more significant than that in high social stratum, which suggests that improving residents' health literacy may be an effective way to alleviate the health gap among different social strata; (2) Health literacy will play an role to improve health outcomes (chronic diseases and self-rated health); (3) There are significant differences in health outcomes among different social strata.

This study also has the following limitations. Firstly, this study is a cross-sectional survey, which can only provide some clues for causal inference between health literacy and health outcomes, but can not verify the causal relationship. Further research is needed to verify the causal relationship. Secondly, the types of health outcomes selected in this study are limited, and it is unknown whether health literacy has the same influence on other health outcomes with social stratum differences. Finally, this paper measures socioeconomic status by simply adding education level, income level and professional status. However, the influences of education level, income level and professional status on socioeconomic status are probably different, so we should further look for a more accurate way to measure socioeconomic status.

## Data Availability

The datasets used and/or analyzed during the current study are available from the corresponding author on reasonable request.

## Footnotes

1 Health literacy level refers to the proportion of persons investigated with health literacy in all persons investigated.
